# TopoICSim: a new semantic similarity measure based on gene ontology

**DOI:** 10.1186/s12859-016-1160-0

**Published:** 2016-07-29

**Authors:** Rezvan Ehsani, Finn Drabløs

**Affiliations:** 1 Department of Cancer Research and Molecular Medicine, Norwegian University of Science and Technology, P.O. Box 8905, NO-7491 Trondheim, Norway; 2 Department of Mathematics, University of Zabol, Zabol, Iran

**Keywords:** Gene ontology, Semantic similarity measure, Tree topology

## Abstract

**Background:**

The Gene Ontology (GO) is a dynamic, controlled vocabulary that describes the cellular function of genes and proteins according to tree major categories: biological process, molecular function and cellular component. It has become widely used in many bioinformatics applications for annotating genes and measuring their semantic similarity, rather than their sequence similarity. Generally speaking, semantic similarity measures involve the GO tree topology, information content of GO terms, or a combination of both.

**Results:**

Here we present a new semantic similarity measure called TopoICSim (Topological Information Content Similarity) which uses information on the specific paths between GO terms based on the topology of the GO tree, and the distribution of information content along these paths. The TopoICSim algorithm was evaluated on two human benchmark datasets based on KEGG pathways and Pfam domains grouped as clans, using GO terms from either the biological process or molecular function. The performance of the TopoICSim measure compared favorably to five existing methods. Furthermore, the TopoICSim similarity was also tested on gene/protein sets defined by correlated gene expression, using three human datasets, and showed improved performance compared to two previously published similarity measures. Finally we used an online benchmarking resource which evaluates any similarity measure against a set of 11 similarity measures in three tests, using gene/protein sets based on sequence similarity, Pfam domains, and enzyme classifications. The results for TopoICSim showed improved performance relative to most of the measures included in the benchmarking, and in particular a very robust performance throughout the different tests.

**Conclusions:**

The TopoICSim similarity measure provides a competitive method with robust performance for quantification of semantic similarity between genes and proteins based on GO annotations. An R script for TopoICSim is available at http://bigr.medisin.ntnu.no/tools/TopoICSim.R.

## Background

### Gene ontology

The Gene Ontology (GO) is a useful resource in bioinformatics that provides structured and controlled vocabularies to describe protein function and localization according to three general categories: biological process (BP), molecular function (MF), and cellular component (CC) [1, 2]. Each of these three annotation categories is structured as its own rooted Directed Acyclic Graph (rDAG). An rDAG is a treelike data structure with a unique root node, the relationships between nodes are directed (oriented), and the structure is non-recursive, i.e. without cycles.

The GO consortium updates on a regular basis a GO Annotation (GOA) [3] database with new GO terms that are linked to genes and gene products by relevant studies. GO is widely used in several bioinformatics applications, including gene functional analysis of DNA microarray data [4], gene clustering [5], disease similarity [6], and prediction and validation of protein-protein interactions [7].

Each GO annotation is assigned together with an evidence code (EC) that refers to the process used to assign the specific GO term to a given gene [8]. All ECs are reviewed by a curator, except ECs assigned with the Inferred from Electronic Annotation (IEA) code.

### Semantic similarity

Measuring similarity between objects that share some attributes is a central issue in many research areas such as psychology, information retrieval, biomedicine, and artificial intelligence [9, 10]. Such similarity measures can be based on comparing features that describe the objects, and a semantic similarity measure uses the relationships which exist between the features of the items being compared [11]. Blanchard et al. have established a general model for comparing semantic similarity measures based on a subsumption hierarchy [12]. They divide tree-based similarities into two categories: those based only on the hierarchical relationships between the terms [13], and those combining additional statistics such as term frequency in a corpus [14].

In a biological perspective, the functional similarity term was proposed to describe the similarity of genes or gene products as the similarity between their GO annotation terms. To establish a suitable functional similarity between genes has become an important aspect of many biological studies. For example have previous studies shown that there is a correlation between gene expression and GO semantic similarity [15].

Since GO terms are organized as an rDAG, the functional similarity can be estimated by a semantic similarity. Pesquita et al. have proposed a general definition of semantic similarity between genes or gene products [16]. Here a semantic similarity is a function which, given two sets of terms annotating two biological entities, returns a numerical value presenting the closeness in meaning between them. This similarity measure is based on comparing all possible pairs of the two sets of GO terms, or selective subsets of them.

### Comparing terms

In general measuring the similarity between two terms can be divided into three main categories: edge-based, node-based and hybrid methods. The edge-based approaches are based on counting the number of edges in the specific path between two terms. In most edge-based measures, a distance function is defined on the shortest path (*SP*) or on the average of all paths [17, 18]. This distance can easily be converted into a similarity measure. Such approaches rely on two assumptions which are seldom true in biological reality. First that nodes and edges are uniformly distributed, and second that edges at the same level in the GO graph correspond to identical distances between terms. Node-based measures are based on the information content (*IC*) of the terms involved. The *IC* value gives a measure of how specific and informative a term is. The *IC* is relying on the probability of terms occurring in a corpus, and Resnik [19] used the negative logarithm of the likelihood of a term to quantify its *IC*.

1 $$ IC(t)=- logp(t) $$

This definition leads to higher *IC* for terms with lower frequency. Obviously, *IC* values increase as a function of depth in the GO graph (this is illustrated in the presentation of TopoICSim, in Results). Resnik used the maximal value among all common ancestors between two terms as a similarity measure, i.e., the *IC* of the lowest common ancestor (LCA) [19]. Since the similarity value of Resnik’s measure is not limited to one (1.0), Lin [14] and Jiang [20] proposed their methods to normalize the similarity value between 0.0 and 1.0. Most node-based methods are based on Resnik’s measure which only considers the *IC* of a single common ancestor and ignores the information on paths in subgraphs composed from common ancestors and pairs GO terms. So, hybrid methods have been proposed to account for both nodes and edges in the subgraph. For example Wang et al. introduced a similarity measure combining the structure of the GO graph with the *IC* values, integrating the contribution of all terms in a GO subgraph, including all the ancestors [21].

### Comparing genes or gene products

Genes are normally annotated using several terms within a particular GO category (MF, BP or CC). Thus, with an available measure function to compute similarity of terms, it is necessary to define an aggregated similarity measure to compare sets of terms. Generally these measures can be divided into two categories: pairwise and groupwise methods [16].

Pairwise approaches measure similarity between two genes by combining the similarities between their terms. Some approaches apply all possible pairwise combination of terms from the two sets, whereas others consider only the best-matching pair for each term. The final similarity between two genes is then defined by combining these pairwise similarities, mostly by the average, the maximum, or the sum [3, 19].

Groupwise methods are not based on combing similarities between individual terms, but rather compute gene similarities by one of three main approaches: set, graph, or vector. In set approaches the similarity is computed by set techniques on the annotations. Graph-based similarity measures calculate similarity between genes using graph matching techniques where each gene is presented as subgraphs of GO terms. And finally, in vector approaches each gene is represented in vector space with each term corresponding to a dimension. Similarity can be estimated using vector-based similarity measures, mostly cosine similarity [22].

### Existing measures

For presentation of some existing methods we introduce the following definitions. Suppose *g*_1_ and *g*_2_ are two given genes or gene products annotated by two sets of GO terms {*t*_11_, *t*_12_, …, *t*_1*n*_} and {*t*_21_, *t*_12_, …, *t*_2*m*_}. The first measure we will introduce is IntelliGO [22], which is a vector-based method. Each gene is represented as a vector *g* = ∑_*i*_*α*_*i*_*e*_*i*_ where *α*_*i*_ = *w*(*g*, *t*_*i*_)*IFA*(*t*_*i*_), and where *w*(*g*, *t*_*i*_) represents the weight assigned to the evidence code between *g* and *t*_*i*_, and *IFA*(*t*_*i*_) is the invers annotation frequency of the term *t*_*i*_. Here *e*_*i*_ is the *i-*th basis vector corresponding to the annotation term *t*_*i*_. The dot product between two gene vectors is defined as in (2) and (3).

2 $$ {g}_1*{g}_2={\displaystyle {\sum}_{\mathrm{i},j}}{\alpha}_i{\beta}_j{e}_i*{e}_j $$

3 $$ {e}_i*{e}_j=\frac{2 Depth(LCA)}{MinSPL\left({t}_{1i},{t}_{2j}\right)+2 Depth(LCA)} $$

Here *Depth*(*LCA*) is the depth of the deepest common ancestor for *t*_1*i*_, *t*_2*j*_ and *MinSPL*(*t*_1*i*_, *t*_2*j*_) is the length of the shortest path between *t*_1*i*_, *t*_2*j*_ which passes through *LCA*. The similarity measure for two genes vectors *g*_1_ and *g*_2_ is then defined using the cosine formula (4).

4 $$ SI{M}_{IntelliGO}\left({g}_1,{g}_2\right) = \frac{g_1*{g}_2}{\sqrt{g_1*{g}_1}\sqrt{g_2*{g}_2}} $$

The second measure presented here was introduced by Wang et al. [21]. They considered for the different contributions that terms are related by *is_a* and *part_of*. The semantic contribution that ancestor terms make to a child term is estimated by (5).

5 $$ SV(t)={\displaystyle \sum_{x\in Anc(t)}}{S}_t(x) $$

Here *S*_*t*_(*t*) = 1 and *S*_*t*_(*x*) = max{*w*_*e*_ * *S*_*t*_(*t*_*i*_)|*t*_*i*_ ∈ *childrenof*(*x*)}, where *w*_*e*_ ∈ [0, 1] is a value that corresponds to the semantic contribution factor for edge *e*, and *childrenof*(*x*) returns the immediate children of *x* that are ancestors of *t* and ${S}_t\left({t}_i\right)={\displaystyle {\prod}_{x\in P\left(t,{t}_{i-1}\right)} max\ {w}_k}$ where *P*(*t*, *t*_*i* − 1_) is the path between *t* and *t*_*i* − 1_*.* They used the weights *w*_*is*_*a*_ = 0.8 and *w*_*part*_*of*_ = 0.6. Then they defined the similarity of two terms as in (6).

6 $$ S\left({t}_{1i},{t}_{2j}\right)=\frac{{\displaystyle {\sum}_{x\in ComAnc\left({t}_{1i},{t}_{2j}\right)}}{S}_{t_{1i}}(x)+{S}_{t_{2j}}(x)}{SV\left({t}_{1i}\right)+SV\left({t}_{2j}\right)} $$

Finally the Wang measure uses a best-matched approach (BMA) to calculate similarity between two genes according to (7).

7 $$ SI{M}_{Wang}\left({g}_1,{g}_2\right)=\frac{\sum_{i=1}^nma{x}_jS\left({t}_{1i},{t}_{2j}\right)+{\sum}_{j=1}^mma{x}_iS\left({t}_{1i},{t}_{2j}\right)}{n+m} $$

The third measure is Lord’s measure [3], which is based on Resnik’s similarity. The Resnik similarity is defined as in (8).

8 $$ SI{M}_{Resnik}\left({t}_{1i},{t}_{2j}\right)=IC\left(LCA\left({t}_{1i},{t}_{2j}\right)\right) $$

The Lord measure is estimated as the average of the Resnik similarity over all *t*_1*i*_ and *t*_2*j*_.

9 $$ SI{M}_{Lord}\left({g}_1,{g}_2\right)=\frac{\sum_{i=1}^n{\sum}_{j=1}^mSI{M}_{Resnik}\left({t}_{1i},{t}_{2j}\right)}{n\times m} $$

The next measure was introduced by Al-Mubaid et al. [23]. First they calculate the length of all shortest paths (*PLs*) for all (*t*_1*i*_, *t*_2*j*_) pairs. Then the average on the *PLs* defines the distance between two genes *g*_1_ and *g*_2_ as in (10).

10 $$ PL\left({g}_1,{g}_2\right)=\frac{\sum_{i=1}^n{\sum}_{j=1}^m PL\left({t}_{1i},{t}_{2j}\right)}{n\times m} $$

Finally they use function (11) to convert the distance to a similarity value.

11 $$ SI{M}_{Mubaid}\left({g}_1,{g}_2\right)={e}^{-0.2\times PL\left({g}_1,{g}_2\right)} $$

The last measure presented here is SimGIC [24], which also is called the Weighted Jaccard measure. Let *G*_1_ and *G*_2_ be the GO terms and their ancestors for two genes *g*_1_ and *g*_2_, respectively. The SimGIC is defined as the ratio between the sum of the *ICs* of terms in the intersection and the sum of the *ICs* of terms in the union (12).

12 $$ SimGIC\left({g}_1,{g}_2\right)=\frac{{\displaystyle {\sum}_{t\in {G}_1{\displaystyle \cap }{G}_2}}IC(t)}{{\displaystyle {\sum}_{t\in {G}_1{\displaystyle \cup }{G}_2}}IC(t)} $$

We will now describe the implementation and testing of a new method, TopoICSim, and compare it to the measures introduced above using several different test data sets. In this measure we have tried to decrease any bias induced by irregularity of the rDAG. In particular, TopoICSim examines all common ancestors for a pair of GO terms, and not only the last (or deepest) common ancestor, which is the case for the measures introduced above. Details regarding the evaluation measures, the datasets and approaches that were used for benchmarking and the actual implementation are given in Methods.

## Methods

### IntraSet similarity and discriminating power

To evaluate TopoICSim relative to existing methods we first used two different benchmarks based on the GO properties studied by Benabderrahmane et al. [22]. For the KEGG benchmark they used a diverse set of 13 human KEGG pathways. The assumption when testing the KEGG dataset is that genes belonging to a specific pathway share a similar biological process, so the estimated similarity was based on BP annotations (Table 1). They also defined a Pfam benchmark, using data from the Sanger Pfam database [25] for 10 different Pfam human clans. The assumption when testing Pfam clans is that genes belonging to a specific clan share a similar molecular function, so the estimated similarity was based on MF annotations (Table 1).

**Table 1:** List of human KEGG pathways and Pfam clans used for benchmarking

	KEGG			Pfam	
Pathway	Name	#genes	Accession	Name	#genes
hsa00040	Pentose and glucuronate interconversions	26	CL0099.10	ALDH-like	18
hsa00920	Sulfur metabolism	13	CL0106.10	6PGD_C	8
hsa00140	C21-Steroid homone metabolism	17	CL0417.1	BIR-like	9
hsa00290	Valine, leucine and isoleucine biosynthesis	11	CL0165.8	Cache	5
hsa00563	Glycosylphosphatidylinositol (GPI)-anchor biosynthesis	23	CL0149.9	CoA-acyltrans	7
hsa00670	One carbon pool by folate	16	CL0085.11	FAD_DHS	12
hsa00232	Caffeine metabolism	7	CL0076.9	FAD_Lum_binding	18
hsa03022	Basal transcription factors	38	CL0289.3	FBD	6
hsa03020	RNA polymerase	29	CL0119.10	Flavokinase	7
hsa04130	SNARE interactions in vesicular transport	38	CL0042.9	Flavoprotein	10
hsa03450	Non-homologous end-joining	14			
hsa03430	Mismatch repair	23			
hsa04950	Maturity onset diabetes of the young	25			
Total #genes		280			100

These datasets were obtained directly from [22]

They used two measures, *IntraSet Similarity* and *Discriminating Power* on the benchmark datasets to evaluate their method. Let *S* be a collection of genes where *S* = {*S*_1_, *S*_2_, …, *S*_*p*_} (each *S*_*k*_ can be e.g. a Pfam clan or a KEGG pathway). For each *S*_*k*_, let {*g*_*k*1_, *g*_*k*2_, …, *g*_*kn*_} be the set of *n* genes in *S*_*k*_. *IntraSet* similarity is a measure to calculate the average similarity over all pairwise comparisons within a set of genes (13).

13 $$ IntraSetSim\left({S}_k\right)=\frac{\sum_{i=1}^n{\sum}_{j=1}^nSim\left({g}_{ki},{g}_{kj}\right)}{n^2} $$

*InterSet* similarity can be estimated for two sets of genes *S*_*k*_ and *S*_*l*_ composed of *n* and *m* genes, respectively, as the average of all similarities between pairs of genes from each of the two sets *S*_*k*_ and *S*_*l*_ (14).

14 $$ InterSetSim\left({S}_k,{S}_l\right)=\frac{\sum_{i=1}^n{\sum}_{j=1}^mSim\left({g}_{ki},{g}_{lj}\right)}{n\times m} $$

The ratio of the *IntraSet* and *InterSet* average gene similarities can be defined as the discriminating power (*DP)* (15).

15 $$ D{P}_{Sim}\left({S}_k\right)=\frac{\left(p-1\right) IntraSetSim\left({S}_k\right)}{\sum_{i=1,i\ne k}^p InterSetSim\left({S}_k,{S}_i\right)} $$

It is important to have high *IntraSet* similarity and at the same time high *Discriminating Power* for a measure. Therefore we decided to define a new measure, *IntraSet Discriminating Power* (*IDP*), using the following formula (16).

16 $$ ID{P}_{Sim}\left({S}_k\right)= IntraSetSim\left({S}_k\right)\times D{P}_{Sim}\left({S}_k\right) $$

The *IDP* value estimates the ability to identify similarity between gene sets in a dataset, and at the same time discriminate these sets from other genes in the dataset.

We compared the results obtained with our TopoICSim method with the five existing state-of-the-art similarity measures described in the introduction. For the benchmark datasets, *IntraSet, DP,* and *IDP* values were calculated by our method and compared to those estimated using the other measures.

### Expression similarity

Many recent studies have shown that genes that are biologically and functionally related often maintain this similarity both in their expression profiles as well as in their GO annotations [15]. To test this assumption we selected three sets of genes from the Hallmark datasets, which is a collection of 50 gene sets representing specific well-defined biological processes [26]. These three gene sets are labeled as G2M_CHECKPOINT, DNA_REPAIR, and IL6_JAK_STAT3_SIGNALING, with 200, 151, and 87 genes respectively. The expression values for the genes across multiple cell types and experiments have been obtained from FANTOM5 [27] using the “CAGE peak based expression table (RLE normalized) of robust CAGE peaks for human samples with annotation” file. The expression values were listed according to clusters of transcriptional start sites, therefore some genes were initially assigned multiple expression values, corresponding to unique clusters of start sites. We combined expression values for each gene and then transformed the total expression by log2. Each gene could then be represented as a vector with 1829 expression values.

We used three expression similarities (Pearson correlation, Spearman correlation, and Distance correlation (*DC*)), against the three annotation similarities (TopoICSim, IntelliGO, and Wang) that showed the best performance during initial testing (see Results).

Previous studies have shown that in most cases there is no meaningful correlation when pairs of individual genes are used to estimate correlation between expression and annotation similarities, but that this can be improved by grouping methods, comparing groups or clusters of genes [15]. In these methods, the gene pairs are split into groups of equal intervals according to the annotation (or expression) similarity values between the gene pairs. Then correlation between expression and annotation similarities is defined as correlation between the average of these similarities on the splits [28, 29]. There are many reasons for poor correlation when interactions between individual genes are considered. For example, genes may be involved in multiple and different processes across a dataset. Comparison of individual genes will underestimate similarity due to these differences, whereas grouping methods can highlight shared properties within groups. We therefore decided to group results by using a Self-Organizing Map (SOM) algorithm on (r, s) pairs, where r and s are one of the expression and annotation similarities respectively. A SOM is a topology-preserving mapping of high-dimensional data based on artificial neural networks. It consists of a geometry of nodes mapped into a k-dimensional space, initially at random, which is iteratively adjusted. In each iteration the nodes move in the direction of selected data points, where the movement depends upon the distances to the data points, so that data points located close to a given node have a larger influence than data points located far away. Thereby, neighboring points in the initial topology tend to be mapped to close or identical nodes in the k-dimensional space [30]. We calculated correlation between expression and annotation similarities for all clusters and then identified clusters showing good correlation. Final correlation is reported as average correlation of individual expression and annotation similarities within these clusters. This approach was applied to all possible combination of (r, s) values, i.e., 9 combinations in total.

### Distance correlation

Distance Correlation (*DC*) as introduced by Székely and Bakirov [31] is a method to estimate the dependency between two random variables. It measures the discrepancy between the joint function and the product of its marginal functions in a specific weighting scheme in *L*_2_ space. More strictly, let (*X*, *Y*) be a pair of random variables with joint function *f*_(*X*, *Y*)_ and marginal functions *f*_*X*_ and *f*_*Y*_. The distance covariance can be defined as the root of the following Eq. (17).

17 $$ dco{v}^2\left(X,\ Y\right)={\displaystyle \int }{\left|{f}_{\left(X,\ Y\right)}\left(t,\ s\right)-{f}_X(t){f}_Y(s)\right|}^2w\left(t,\ s\right) dtds $$

This is on *R*^*p* + *q*^ where *p* and *q* are the dimension of *X* and *Y* respectively and *w*(*t*, *s*) is the weight function. Now, the *DC* can be defined by distance covariance as in (18).

18 $$ dcor\left(X,\ Y\right)=\frac{dcov\left(X,Y\right)}{\sqrt{dcov\left(X,\ X\right)}\sqrt{dcov\left(Y,\ Y\right)}} $$

It has been shown that the empirical *DC* for an iid sample {(*x*_1_, *y*_1_), (*x*_2_, *y*_2_), …, (*x*_*n*_, *y*_*n*_)} can be estimated as in (19–22).

19 $$ DC\left(X,Y\right)={S}_1+{S}_2-2{S}_3 $$

20 $$ {S}_1=\frac{1}{n^2}{\displaystyle \sum_{k,l=1}^n}{\left|{x}_k-{x}_l\right|}_p{\left|{y}_k-{y}_l\right|}_q $$

21 $$ {S}_2=\frac{1}{n^2}{\displaystyle \sum_{k,l=1}^n}{\left|{x}_k-{x}_l\right|}_p\frac{1}{n^2}{\displaystyle \sum_{k,l=1}^n}{\left|{y}_k-{y}_l\right|}_q $$

22 $$ {S}_3=\frac{1}{n^3}{\displaystyle \sum_{k=1}^n}\ {\displaystyle \sum_{l,m=1}^n}{\left|{x}_k-{x}_l\right|}_p{\left|{y}_k-{y}_m\right|}_q $$

Some previous studies have applied *DC* on the expression level of gene sets [32, 33].

### Evaluation by CESSM

Collaborative Evaluation of GO-based Semantic Similarity Measures (CESSM) is an online tool [34] that enables the comparison of a given measure against 11 previously published measures based on their correlation with sequence, Pfam, and Enzyme Classification (ECC) similarities [35]. It uses a dataset of 13,430 protein pairs involving 1,039 unique proteins from various species. Protein pairs (from multiple species), GO (dated August 2010), and UniProt GO annotations (dated August 2008) were downloaded from CESSM. The similarities for the 13,430 proteins pairs were calculated with TopoICSim and returned to CESSM for evaluation.

### Implementation

The R programming language (version 3.2.2) was used for developing and running all programs. We used all the EC codes as annotation terms. The *ppiPre* (version 1.9)*, GeneSemSim* (version 1.28.2)*,* and *csbl.go* (version 1.4.1) packages were used to calculate IntelliGO, Wang, and SimGIC measures [36–38]. The *DC* values were estimated using the *energy* (version 1.6.2) package [39]. The SOM algorithm was performed with the *SOMbrero* (version 1.1) package [40]. All these packages are available within R Bioconductor [41].

## Results

### The TopoICSim measure

Here we introduce a new similarity measure which accounts for the distribution of IC on both shortest path between two terms and longest path from their common ancestor to root. A weighting scheme in terms of length of the paths is used to provide a more informative similarity measure. In the current version we do not use any weight scheme on the ECs codes. We use definitions of relevant concepts as follows.

A GO tree can be described as a triplet Λ = (*G*, Σ, *R*), where *G* is the set of GO terms, Σ is the set of hierarchical relations between GO terms (mostly defined as *is_a* or *part_of*) [22], and *R* is a triplet (*t*_*i*_, *t*_*j*_, *ξ*), where *t*_*i*_, *t*_*j*_ ∈ *G* and *ξ* ∈ *R* and *t*_*i*_*ξt*_*j*_. The *ξ* relationship is an oriented child–parent relation. Top level node of the GO rDAG is the Root, which is a direct parent of the MF, BP, and CC nodes. These nodes are called aspect-specific roots and we refer to them as *root* in following.

A path *P* of length *n* between two terms *t*_*i*_, *t*_*j*_ can be defined as in (23).

23 $$ P:G\times G\to G\times G\cdots \times G={G}^{n+1};\kern0.28em P\left({t}_i,{t}_j\right)=\left({t}_i,{t}_{i+1},\dots, {t}_j\right) $$

Here ∀ *s*, *i* ≤ *s* < *j*, ∃ *ξ*_*s*_ ∈ Σ, ∃ *τ*_*s*_ ∈ *R*, *τ*_*s*_ = (*t*_*s*_, *t*_*s* + 1_, *ξ*_*s*_). Because *G* is an rDAG, there might be multiple paths between two terms, so we represent all paths between two terms *t*_*i*_, *t*_*j*_ according to (24).

24 $$ \mathcal{A}\left({t}_i,{t}_j\right)={\displaystyle \underset{P}{\cup }P}\left({t}_i,{t}_j\right) $$

We use Inverse Information Content (*IIC*) values to define shortest and longest paths for two given terms *t*_*i*_, *t*_*j*_ as shown in (25–27).

25 $$ SP\left({t}_i,{t}_j\right)=\underset{P\in A\left({t}_i,{t}_j\right)}{\mathrm{argmin}}IIC(P) $$

26 $$ LP\left({t}_i,{t}_j\right)=\underset{P\in A\left({t}_i,{t}_j\right)}{\mathrm{argmax}}IIC(P) $$

27 $$ IIC(P)={\displaystyle \sum_{t\in P}}\frac{1}{IC(t)} $$

We used a standard definition to calculate *IC*(*t*) as shown in (28)

28 $$ IC(t)=- \log \frac{G_t}{G_{Tot}} $$

Here *G*_*t*_ is the number of genes annotated by the term *t* and *G*_*Tot*_ is the total number of genes. The distribution of *IC* is not uniform in the rDAG, so it is possible to have two paths with different lengths but with same *IICs*. To overcome this problem we weight paths by their lengths, so the definitions in (25) and (26) can be updated according to (29) and (30).

29 $$ wSP\left({t}_i,{t}_j\right)=SP\left({t}_i,{t}_j\right)\times len(P) $$

30 $$ wLP\left({t}_i,{t}_j\right)=LP\left({t}_i,{t}_j\right)\times len(P) $$

Now let *ComAnc*(*t*_*i*_, *t*_*j*_) be the set of all common ancestors for two given terms *t*_*i*_, *t*_*j*_. First we define the disjunctive common ancestors as a subset of *ComAnc*(*t*_*i*_, *t*_*j*_) as in (31).

31 $$ DisComAnc\left({t}_i,{t}_j\right) = \left\{x\in ComAnc\left({t}_i,{t}_j\right)\ \Big|\ P\left(x, root\right)\cap C(x)=\varnothing \right\} $$

Here *P*(*x*, *root*) is the path between *x* and *root* and *C*(*x*) is set of all immediate children for *x*.

For each disjunctive common ancestor *x* in *DisComAnc*(*t*_*i*_, *t*_*j*_), we define the distance between *t*_*i*_, *t*_*j*_ as the ratio of the weighted shortest path between *t*_*i*_, *t*_*j*_ which passes from *x* to the weighted longest path between *x* and *root*, as in (32–33).

32 $$ D\left({t}_i,{t}_j,x\right)=\frac{wSP\left({t}_i,{t}_j,x\right)}{wLP\left(x, root\right)} $$

33 $$ wSP\left({t}_i,{t}_j,x\right)=wSP\left({t}_i,x\right)+wSP\left({t}_j,x\right) $$

Now the distance for two terms *t*_*i*_, *t*_*j*_ can be defined according to (34).

34 $$ D\left({t}_i,{t}_j\right)=\underset{x\in DisComAnc\left({t}_i,{t}_j\right)}{ \min }D\left({t}_i,{t}_j,x\right) $$

We convert distance values by the $$ \frac{Arctan(.)}{\raisebox{1ex}{$\pi $}\!\left/ \!\raisebox{-1ex}{$2$}\right.} $$ function, and the measure for two GO terms *t*_*i*_ and *t*_*j*_ can be defined as in (35).

35 $$ S\left({t}_i,{t}_j\right)\kern0.5em =\kern0.5em 1-\frac{Arctan\left(D\left({t}_i,{t}_j\right)\right)}{\raisebox{1ex}{$\pi $}\!\left/ \!\raisebox{-1ex}{$2$}\right.} $$

Note that *root* refers to one of three first levels in the rDAG. So if *DisComAnc*(*t*_*i*_, *t*_*j*_) = {*root*} then *D*(*t*_*i*_, *t*_*j*_) = ∞ and *S*(*t*_*i*_, *t*_*j*_) = 0. Also if *t*_*i*_ = *t*_*j*_ then *D*(*t*_*i*_, *t*_*j*_) = 0 and *S*(*t*_*i*_, *t*_*j*_) = 1.

Finally let *S* = [*s*_*ij*_]_*n* × *m*_ be a similarity matrix for two given genes or gene products *g*_1_, *g*_2_ with GO terms {*t*_11_, *t*_12_, …, *t*_1*n*_} and {*t*_21_, *t*_12_, …, *t*_2*m*_}, where *s*_*ij*_ is the similarity between the GO terms *t*_1*i*_ and *t*_2*j*_. We use a *rcmax* method to calculate similarity between *g*_1_, *g*_2_, as defined in (36).

36 $$ TopoICSim\left({g}_1,{g}_2\right)= rcmax(S)= max\left(\frac{\sum_{i=1}^n\underset{j}{ \max }{s}_{ij}}{n},\ \frac{\sum_{j=1}^m\underset{i}{ \max }{s}_{ij}}{m}\right) $$

We also tested other methods on the similarity matrix, in particular average and BMA, but in general *rcmax* gave the best performance for TopoICSim (data not shown).

### The TopoICSim algorithm

**Fig. 1:**
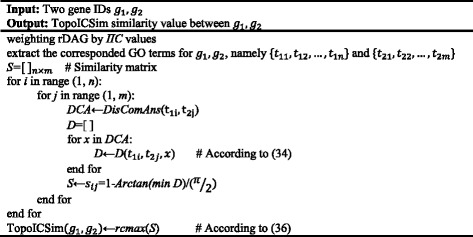
Pseudocode for the TopoICSim algorithm

The TopoICSim algorithm was implemented to estimate the similarity between two genes, taking their gene ID (currently *Entrez ID*) as input, together with parameters: a GO annotation type (MF, BP, and CC), a species, and an EC specification (default is NULL, which means using all ECs). The output is the similarity between the two genes. Pseudocode for the TopoICSim algorithm is presented in Fig. 1.

The *IC*s used to weigh the GO terms were calculated using the GOSim package (version 1.8.0) [42]. For each disjunctive the shortest path between the two GO terms was calculated by the *Dijkstra* algorithm in the RBGL package (version 1.46.0) [43] according to (25). Also the longest path between the disjunctive and *root* was calculated by the *topology sorting* algorithm [44] according to (26).

#### A simple example

**Fig. 2:**
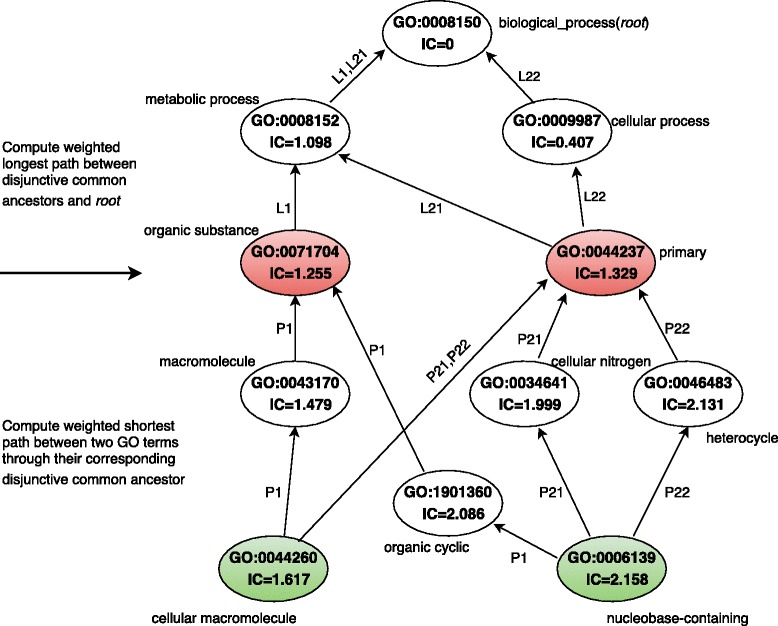
Sample GO structure illustrating the main computations used in TopoICSim

To exemplify how TopoICSim computes the similarity between two given GO terms, we will illustrate the similarity between the two GO terms GO:0044260 and GO:0006139 as shown in Fig. 2, using the BP ontology of GO. According to (32), these GO terms have two disjunctive ancestors: GO:0071704 and GO:0044237. For GO:0071704 there are unique paths from GO:0071704 to root and from GO:0044260 and GO:0006139 to GO:0071704 (L1 and P1 in Fig. 2 respectively). Therefore, according to (32) the distance between these GO terms will be:

$$ D\left({\textstyle \mathrm{GO}:0044260},{\textstyle \mathrm{GO}:0006139},{\textstyle \mathrm{GO}:0071704}\right)=\frac{\left(\frac{1}{2.158}+\frac{1}{2.086}+\frac{1}{1.255}+\frac{1}{1.479}+\frac{1}{1.617}\right)\times 4}{\left(\frac{1}{1.255}+\frac{1}{1.098}\right)\times 2}=2.75 $$

For GO:0044237 there are two paths from GO:0044237 to root (L21 and L22) and two paths from GO:0044260 and GO:0006139 to GO:0044237 (P21 and P22). According to (25) and (26) and the IC values in Fig. 2 L22 and P22 are longest path and shortest path respectively, so distance for this case will be:

$$ D\left({\textstyle \mathrm{GO}:0044260},{\textstyle \mathrm{GO}:0006139},{\textstyle \mathrm{GO}:0044237}\right)\kern0.5em =\kern0.75em \frac{\left(\frac{1}{2.158}+\frac{1}{1.999}+\frac{1}{1.329}+\frac{1}{1.617}\right)\times 3}{\left(\frac{1}{1.329}+\frac{1}{0.407}\right)\times 2}\kern0.5em =\kern0.5em 1.076 $$

Obviously the second value is the minimum, so the similarity between GO:0044260 and GO:0006139 according to (35) will be:

$$ S\left({\textstyle \mathrm{GO}:0044260},{\textstyle \mathrm{GO}:0006139}\right)=\kern0.5em 1\kern0.5em -\frac{Arctan(1.076)}{\pi /2\operatorname{}}=0.477 $$

### Benchmarking of TopoICSim

With the growing number of similarity measures, an important issue is comparison of their performance. For this, in particular the five similarity measures presented in the introduction were considered for comparison with TopoICSim in several tests.

### IntraSet similarity

**Fig. 3:**
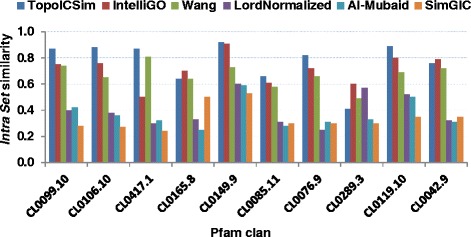
*IntraSet* similarities for the Pfam clan dataset using MF annotations. The *IntraSet* similarity is estimated for all pairs of genes within in each clan using MF annotations over all considered similarity measures

**Fig. 4:**
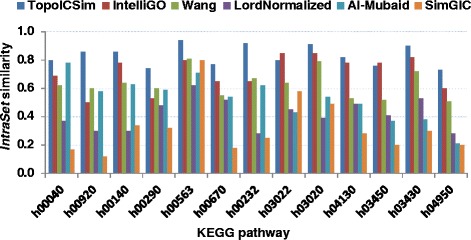
*IntraSet* similarities for KEGG pathways dataset using BP annotations. The *IntraSet* similarity is estimated for all pair genes within each KEGG pathway using BP annotations for all considered similarity measures

The *IntraSet* similarity is the average similarity over all pairwise comparisons within a set of genes. The *IntraSet* values were calculated with TopoICSim and five other algorithms, namely IntelliGO, Wang, Lord-normalized, Al-Mubaid, and SimGIC, using data sets defined by Pfam clans and KEGG pathways. The performance results obtained with the Pfam clans using MF annotations are shown in Fig. 3. For 7 out of 10 Pfam clans, the TopoICSim measure showed generally higher *IntraSet* similarity compared to the other measures, and only for the CL0289.3 case did it show lower performance. The results for the KEGG pathway datasets based on BP annotations were very similar (Fig. 4). Again the TopoICSim measure had in general higher performance compared to the other measures (11 out of 13).

### Discriminating power

**Fig. 5:**
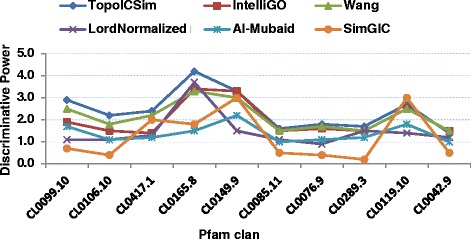
Comparison of the discriminating power of six similarity measures using Pfam clan and MF annotations. The discriminating power values estimated using all considered similarity measures are plotted for all Pfam clans

**Fig. 6:**
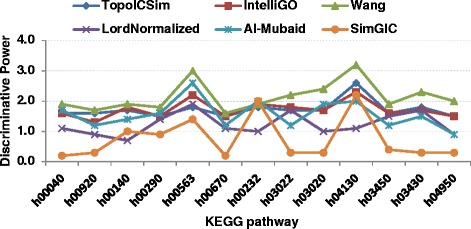
Comparison of the discriminating power of six similarity measures using KEGG pathway and BP annotations. The discriminating power values estimated with all considered similarity measures are plotted for all KEGG pathways

The Discriminating Power (*DP*) is defined as the ratio of the *IntraSet* and *InterSet* average gene similarities, where *InterSet* similarities are between gene sets, rather than within. The calculated *DP* values for all methods on the two benchmark datasets used for *IntraSet* similarity are plotted in Figs. 5 and 6. For the Pfam Clans and MF annotations TopoICSim measure was superior compared to the other methods. The minimum and maximum *DP* values generated by the TopoICSim were 1.4 for CL0042.9 and 4.2 for CL0165.8, respectively. For the KEGG pathway dataset the Wang measure provide better performance compared to IntelliGO and TopoICSim, which came second and third.

### IntraSet discriminating power

**Fig. 7:**
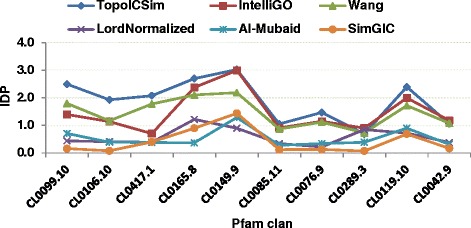
Comparison of the *IDP* values of six similarity measures using Pfam clan and MF annotations

**Fig. 8:**
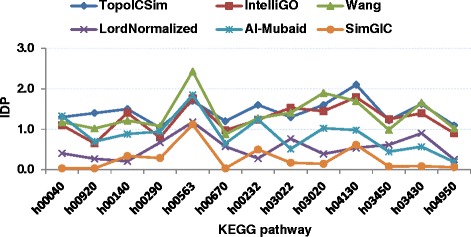
Comparison of the *IDP* values of six similarity measures using KEGG pathways and BP annotations

*IntraSet* Discriminating Power (*IDP*) represents a combination of the *InstraSet* similarity and *DP*, as both should be high for an optimal measure. The *IDP* values were estimated for all measures in the study using formula (16). The results are plotted in Figs. 7 and 8 for MF and BP annotations respectively. For the MF annotations for Pfam clan data TopoICSim shows a generally better performance compared to the other measures. For the BP annotations for KEGG pathway data the best performance was seen for the TopoICSim, IntelliGO, and Wang measures. The TopoICSim had best performance (unique or shared best) for 10 out of 13 cases. It therefore shows a very good and robust performance in this part of the evaluation.

### Evaluation versus expression similarity

For evaluation of TopoICSim with respect to annotation similarity associated with expression similarity we used three subsets of human genes from [45], namely G2M, DNA_REPAIR, and STAT3. For each subset both expression and annotation similarities were calculated using Pearson and Spearman correlations and *DC* for expression similarity based on CAGE data (see Methods) (r values), and TopoICSim, IntelliGO, and Wang for semantic similarity (s values). The Self-Organizing Map (SOM) algorithm was used to cluster all interactions into three subsets based on (r, s) values. A 6 × 6 square topology was selected to set up the SOM computation. The correlation was computed for each cluster and the clusters with *r* > =0.5 were used to estimate final correlation between expression and annotation similarities as an average on the correlation values within these selected clusters. Table 2 presents the correlation values for each of the three subsets and the considered (r, s) pairs. For the three sets of genes that were tested the maximum correlation was seen when we used the *DC* correlation and TopoICSim measures for the expression and annotation similarities (0.943, 0.921, and 0.890 for G2M, DNA_REPAIR, and STAT3 respectively). Also, the calculated correlations with the TopoICSim measure were higher than the correlation values calculated by the two other measures for all cases except the DNA_REPAIR set when using the Spearman and IntelliGO combination (0.89).

**Table 2:** Correlation between expression and annotation similarities

		G2M			DNA_REPAIR			STAT3	
	TopoICSim	IntelliGO	Wang	TopoICSim	IntelliGO	Wang	TopoICSim	IntelliGO	Wang
Pearson	0.932	0.572	0.849	0.890	0.879	0.867	0.833	0.795	0.824
Spearman	0.914	0.548	0.871	0.876	0.890	0.813	0.872	0.766	0.793
DC	**0.943**	0.594	0.885	**0.921**	0.887	0.863	**0.890**	0.801	0.827

Numbers in bold indicate the best correlation for each subset when comparing TopoICSim, IntelliGO and Wang

### Evaluation by CESSM

The TopoICSim measure was used to calculate similarities for the benchmark set of protein pairs downloaded from the CESSM website [34]. The benchmark set represents three different types of similarities, based on sequence similarity (SeqSim), enzyme classification (ECC), and protein domains (Pfam). The results obtained (correlation coefficients) are presented in Table 3. When we used the MF annotations, the correlation coefficients range from 0.55 for the SeqSim dataset to 0.75 for the ECC dataset. The TopoICSim correlation coefficient for the ECC dataset is higher than all other methods. For the Pfam dataset TopoICSim is at a similar level as SimGIC (0.62 vs. 0.63). For the SeqSim dataset the value obtained with TopoICSim is beaten by four other methods (SimGIC, SimUI, RB, LB).

**Table 3:** Results obtained with the CESSM benchmarking tool

Metrics								Methods					
		SimGIC	SimUI	RA	RM	RB	LA	LM	LB	JA	JM	JB	TopoICSim
MF	ECC	0.62	0.63	0.39	0.45	0.60	0.42	0.45	0.64	0.34	0.36	0.56	**0.75**
	Pfam	**0.63**	0.61	0.44	0.18	0.57	0.44	0.18	0.56	0.33	0.12	0.49	0.62
	SeqSim	**0.71**	0.59	0.50	0.12	0.66	0.46	0.12	0.60	0.29	0.10	0.54	0.55
BP	ECC	0.39	0.40	0.30	0.30	0.44	0.30	0.31	0.43	0.19	0.25	0.37	**0.46**
	Pfam	0.45	0.45	0.32	0.26	0.45	0.28	0.20	0.37	0.17	0.16	0.33	**0.51**
	SeqSim	**0.77**	0.73	0.40	0.30	0.73	0.34	0.25	0.63	0.21	0.23	0.58	0.68

Pearson correlation coefficients are shown for the ECC, Pfam, and SeqSim datasets. The MF and BP annotations are used. Numbers in bold show the best correlation for each dataset. The column headings represent the following methods: *SimGIC* Similarity Graph Information Content, *SimUI* Union Intersection similarity, *RA* Resnick Average, *RM* Resnick Max, *RB* Resnick Best match, *LA* Lord Average, *LM* Lord Max, *LB* Lord Best match, *JA* Jaccard Average, *JM* Jaccard Max, *JB* Jaccard Best match

For the BP annotations, the performance was generally higher than for MF annotations. For the ECC and Pfam datasets the TopoICSim correlation coefficients are higher than for any of the other measures. For the SeqSim dataset the score obtained by TopoICSim is beaten by three other measures (SimGIC, SimUI, and RB).

### Annotation length bias

Annotations are not uniformly distributed among the genes or gene products within an annotation corpus, and some studies have indicated a clear correlation between semantic scores and the number of annotations [46]. Wang et al. [47] used randomly selected pairs of term groups to evaluate the increase in protein semantic similarity score that resulted only from the increased annotation length, regardless of other biological factors. First, they randomly selected 10,000 pairs of term groups with the same sizes (corresponding to the annotation lengths of proteins) ranging from 1 to 10. Then, using each of 14 semantic similarity scores, they calculated the semantic similarity scores for random term group pairs, and analyzed whether these scores increased as the group size increased using the Spearman rank correlation coefficient. All the 14 semantic similarity methods tested by Wang et al. showed a perfect or close to perfect Spearman correlation (r from 0.99 to 1.00, *p*-value from 9.31e-08 to <2.20e-16). We used their approach and got a Spearman correlation of *r* = 0.70 with *p*-value = 0.02. Although there still is a significant correlation, it is smaller than all reported correlations in Wang et al.

### The shallow annotation problem

Genes that are annotated at only very shallow levels (for example “binding”) can lead to very high semantic similarities [46]. For example, consider the two human genes Akap1 (A-kinase anchor protein 1 – ID:8165) and Bbs9 (Bardet-Bieddl syndrome 9 – ID:27241). The first gene is a trans-membrane protein that has 10 GO terms associated with the MF ontology. The second gene is poorly understood and has only two GO terms, including GO:0005515 (protein binding), which it happens to share with Akap1. Despite this weak link, some node based methods like Lin and Jiang not only predict high similarity, but actually return a maximum score (1.0). The similarity of these genes according to IntelliGO and Wang is 0.763 and 0.643, respectively, whereas TopoICSim generates a more appropriate low similarity of 0.5.

### Running time

Table 4 shows the running times for TopoICSim compared to IntelliGO and Wang, using calculation of the similarity values of all gene pairs in three gene sets that were used for benchmarking*.* It is not surprising that the Wang method has very short running times compared to TopoICSim and IntelliGO, as Wang does not spend time on finding longest and shortest paths. However, the results also show that TopoICSim actually has shorter running time than IntelliGO in each of the tree cases.

**Table 4:** Running time

		Running time (min)		
Gene set	Interactions	TopoICSim	IntelliGO	Wang
STAT3	7569	112	132	15
DNA_REPAIR	22801	312	426	45
G2M	40000	595	815	83

Running times in minutes for calculating similarities over all genes pairs in each of the gene sets

## Discussion

Semantic similarity measures rely upon the quality and completeness of their assigned ontology and annotation corpus. The irregular nature of GO annotation data, for example variable edge lengths (edges at the same level can have different semantic measure), variable depth (terms at the same level can have different level of detail), and variable node density (some areas of the ontology have a larger density of terms than others) should be taken into account by semantic similarity measures.

Most existing methods use in the first step the last (deepest) common ancestor to define similarity between two GO terms, which does not guarantee the shortest path between terms that pass from this common ancestor (i.e. a common ancestor located at a higher level leads to a shorter path between the terms). To overcome this issue TopoICSim measures similarity between two GO terms for all disjunctive common ancestors with the described criteria, and the final similarity measure is returned as the best among them according to (34). Although there are other studies that use disjunctive common ancestors [48], they are node based methods that only use shared information on the disjunctive common ancestors and they do not deal with optimal paths in a subgraph of nodes. Another advantage of the TopoICSim measure is the weighting scheme, which is used according to (29, 30). It leads to a better ability to distinguish between terms with the same semantic similarity but at different levels.

Various strategies have been applied to test the validity of semantic similarity measures [16]. For example, in a gene product interaction network, a functional module is a set of interacting gene products that share a biological process or pathway [46]. Based on this they should display similar MF or BP annotations. This hypothesis was tested by Lord et al. by estimating the correlation between gene annotation (MF annotation) and sequence similarity in set of human proteins [3], since sequence similarity often is associated with functional similarity. Also Guo et al. performed an analysis on all pairs of proteins belonging to the same pathway, which showed higher similarity scores than expected when using BP annotation [49].

For evaluation of the TopoICSim similarity measure in this paper, two benchmarking datasets based on KEGG pathways and Pfam clans were used. These datasets have been obtained directly from [22]. The *IntraSet* similarity, Discriminating Power, and *IntraSet* Discriminating Power values were used for the evaluation. For all quality measures used to evaluate the estimated semantic similarity for these two benchmarking data sets TopoICSim had the best result, except for *DP* values for the KEGG dataset where the Wang method had best performance.

Another common scenario for testing the validity of semantic similarity measures is by testing their correlation with gene expression data. Two gene products with similar function are more likely to have similar expression profile and share same or similar GO terms. Therefore a correlation between gene expressions of two gene products versus the semantic similarity measures can be used as a performance test. Wang et al. [50] compared semantic similarity to expression profile correlation for pairs of genes from the Eisen dataset [51]. They showed that for all the considered measures, high semantic similarity is associated with high expression correlation. Also Sevilla et al. showed correlation between semantic similarity and expression profile, but they dramatically improved it by using grouped data [15]. We took this one step further by applying a SOM algorithm to clustering of gene products by expression and semantic similarities to select clusters with high correlation. The TopoICSim was superior on the three tested datasets compared to all other similarity measures. Finally, the evaluation with CESSM showed that the TopoICSim is a competitive measure relative to SimGIC, which is superior to all other similarity measures in the CESSM test. However, in the other tests SimGIC had a more variable and sometimes very low performance, which means that TopoICSim in general is a more robust similarity measure with a very good overall performance.

The robust performance was confirmed when we tested for annotation length bias, which has been identified as a potential problem for semantic similarity methods [46]. The analysis showed that although the score still showed some dependency on the number of annotations, the dependency in TopoICSim was clearly lower than for other semantic similarity methods that have been tested. Another potential problem is related to shallow annotation, where high-level GO terms may lead to an overestimation of the similarity between genes. Here TopoICSim should be more robust to such bias than most other methods, due to its design. We have illustrated this with a simple example. Finally, a benchmarking of running time for TopoICSim showed good performance compared to IntelliGO.

## Conclusions

In this study we present an improved method for semantic similarity which counts distribution of *IC* on the shortest paths between GO terms and longest path from root to the common ancestors, weighted by their lengths. Several strategies were applied to evaluate the TopoICSim similarity measure. Our results show that the TopoICSim similarity measure is robust, in particular since it was among best similarity measures in all benchmarking tests performed here.

## Abbreviations

BP, biological process; CC, MF, cellular component; DC, distance correlation; DP, discriminating power; EC, evidence code; GO, gene ontology; GOA, gene ontology annotation; IC, information content; LCA, lowest common ancestor; MF, molecular function; rDAG, rooted directed acyclic graph; SOM, self-organizing map; SP, shortest path
